# Lipids, lipoprotein distribution and depressive symptoms: the Multi-Ethnic Study of Atherosclerosis

**DOI:** 10.1038/tp.2016.232

**Published:** 2016-11-29

**Authors:** K L Ong, M J Morris, R L McClelland, J Maniam, M A Allison, K-A Rye

**Affiliations:** 1 School of Medical Sciences, University of New South Wales Australia, Sydney, NSW, Australia; 2 Department of Biostatistics, University of Washington, Seattle, WA, USA; 3 Department of Family Medicine and Public Health, University of California San Diego, La Jolla, CA, USA

## Abstract

Previous studies suggest lower concentrations of total and high-density lipoprotein (HDL) cholesterol to be predictive of depression. We therefore investigated the relationship of lipids and lipoprotein distribution with elevated depressive symptoms (EDS) in healthy men and women from the Multi-Ethnic Study of Atherosclerosis (MESA). Participants were followed up over a 9.5-year period. EDS were defined as a Center for Epidemiological Studies Depression (CES-D) score ⩾16 and/or use of antidepressant drugs. Lipoprotein distribution was determined from plasma using nuclear magnetic resonance spectroscopy. Among 4938 MESA participants (mean age=62 years) without EDS at baseline, 1178 (23.9%) developed EDS during follow-up. In multivariable Cox regression analyses, lower total, low-density lipoprotein (LDL) and non-HDL cholesterol concentrations at baseline were associated with incident EDS over 9.5 years (hazards ratio (HR)=1.11–1.12 per s.d. decrease, all *P*<0.01), after adjusting for demographic factors, traditional risk factors including LDL cholesterol, HDL cholesterol and triglycerides. Lipoprotein particle subclasses and sizes were not associated with incident EDS. Among participants without EDS at both baseline and visit 3, a smaller increase in total or non-HDL cholesterol between these visits was associated with lower risk of incident EDS after visit 3 (HR=0.88–0.90 per s.d. decrease, *P*<0.05). Lower baseline concentrations of total, LDL and non-HDL cholesterol were significantly associated with a higher risk of incident EDS. However, a short-term increase in cholesterol concentrations did not help to reduce the risk of EDS. Further studies are needed to replicate our findings in cohorts with younger participants.

## Introduction

Previous studies have suggested a possible relationship between dyslipidemia and depression. In a study of 29 133 men aged 50–69 years in Finland with a follow-up period of 5–8 years, a low serum total cholesterol concentration at baseline was associated with major depression and death from suicide.^1^ However, this study assessed total and high-density lipoprotein (HDL) cholesterol concentrations only. In another recent study of 2187 Australian men aged 81.6 (s.d. 3.6) years, a lower plasma HDL cholesterol concentration, but not low-density lipoprotein (LDL) cholesterol concentration, was associated with higher risk of depression after 5 years.^2^ These studies are limited by their analysis of data in men only, and a lack of comprehensive assessment of different lipid measures.

As the precise link between lipid profile and depression is not well established, longitudinal studies in other settings are needed to strengthen the evidence for a causal relationship. Moreover, it is not known whether there is any difference in such a relationship across subgroups of subject characteristics, especially race/ethnicity, sex and baseline cholesterol concentration.

As lipoprotein particle subclass and size have been demonstrated to affect cardiovascular risk independent of conventional measures of plasma lipids (such as total cholesterol, HDL cholesterol, LDL cholesterol and triglyceride concentrations), it is possible that lipoprotein distribution may also be related to depression, independent of plasma cholesterol concentrations. Therefore, in the current study we investigated the longitudinal relationship of plasma lipid concentrations and lipoprotein distribution at baseline with the development of elevated depressive symptoms (EDS) in apparently healthy participants from the Multi-Ethnic Study of Atherosclerosis (MESA).

## Materials and methods

### Participants

The MESA study is a multicenter, community-based cohort study on the prevalence, correlates and progression of subclinical cardiovascular disease (CVD). It consists of 6814 men and women of four major ethnic groups (Caucasian, African American, Hispanic American and Chinese American) who were 45–84 years old and free of clinically apparent CVD at baseline.^3^ They were recruited from six US communities (Baltimore, MD; Chicago, IL; Forsyth County, NC; Los Angeles County, CA; New York, NY; and St Paul, MN) between July 2000 and August 2002. Each field site was recruited from locally available sources, which included lists of residents, lists of dwellings and telephone exchanges. In the last few months of the recruitment period, supplemental sources (lists of Medicare beneficiaries from the Centers for Medicare and Medicaid Services and referrals by participants) were used to ensure inclusion of adequate numbers of minority and elderly subjects. Although the cohort was community-based, sampling was performed in a manner that provided balanced recruitment across strata defined by gender, ethnicity and age group (45–54, 55–64, 65–74 and 75–84 years) and was not designed to represent the demographic distribution of the source communities. Selection from the sampling frames differs by site. In three Field Centers (Forsyth County, New York, Chicago), random samples, stratified by age and gender, were selected from the sampling frames. In the others (St Paul, Baltimore, Los Angeles) the sampling frame did not contain demographic information and recruitment was along geographic boundaries (St Paul, Baltimore) or by random digit dialing (Los Angeles) to target areas. Participant exclusion criteria included age <45 or >84 years, physician-diagnosed CVD, current atrial fibrillation, having undergone procedures related to CVD, active treatment for cancer, pregnancy, any serious medical conditions that would prevent long-term participation, weight >300 pounds, cognitive inability, living in or waiting for a nursing home, plans to leave the community within 5 years, language barrier and chest CT scan in the past year. Participants were followed up in person at four clinic visits over a follow-up period of 8.0–11.4 years (mean=9.5 years). The study was approved by the institutional review boards at all participating centers and informed written consent was obtained from all participants. The study was performed in compliance with the principles of the Declaration of Helsinki. Details of the study objectives, design and protocol have been described previously^3^ and are available at http://www.mesa-nhlbi.org.

Among 6814 participants at baseline, valid data on EDS at both baseline and follow-up were available on 6124 participants, of whom 5018 participants did not have EDS at baseline. Out of the 5018 participants, 5000 had their lipoprotein profile measured by nuclear magnetic resonance spectroscopy. After further excluding participants with missing data on conventional measurement of lipids (for example, LDL cholesterol, HDL cholesterol and triglycerides), a total of 4938 participants were included in the analysis.

### Assessment of depressive symptoms

Using the Center for Epidemiological Studies Depression Scale (CES-D), depressive symptoms were assessed by a self-report at visits 1, 3, 4 and 5. CES-D is a 20-item questionnaire developed to assess depressive symptoms in community.^4^ The CES-D items represent the major components of depression and include depressed mood, feelings of worthlessness, feelings of hopelessness, loss of appetite, poor concentration and sleep disturbance. Although the CES-D is not an assessment of clinical depression, a CES-D score ⩾16 has been found to be consistent with at least mild-to-moderate depression or dysthymia.^5^ Data on the use of antidepressant drugs (tricyclics, nontricyclics and monoamine oxidase inhibitors) were also collected in visits 1–5. In this study, EDS was defined as a CES-D score ⩾16 and/or use of antidepressant drugs (as described previously in other MESA studies).^6, 7^ In a sensitivity analysis, we repeated the analysis with EDS defined as a CES-D score of (a) ⩾21 or self-reported use of antidepressant medications and (b) ⩾16 only.^6^ A CES-D score of ⩾21 has been considered as a clinical cut-point to indicate probable major depression.^8^

### Conventional measures of lipids

Venous blood samples were collected after a 12-h fast by certified technicians using standardized venipuncture procedures. Samples were then centrifuged at 2000 *g* for 15 min at 4 °C within 30 min of collection. EDTA plasma samples were aliquoted on ice, stored at −70 °C and then shipped on dry ice to the MESA central laboratory for measurement of lipid levels. HDL cholesterol was measured using the cholesterol oxidase method (Roche Diagnostics, Indianapolis, IN, USA) after precipitation of non-HDL cholesterol with magnesium/dextran sulfate. Triglyceride concentrations were measured using a glycerol-blanked enzymatic method with the Triglyceride GB reagent (Roche Diagnostics) on the Roche COBAS FARA centrifugal analyzer. The laboratory coefficient of variations (CVs) for total cholesterol, HDL cholesterol and triglycerides were 1.6%, 2.9% and 4.0%, respectively. In plasma samples having a triglyceride value <400 mg dl^−1^, LDL cholesterol was calculated using the Friedewald formula.^9^ The two atherogenic indices, total/HDL cholesterol ratio and LDL/HDL cholesterol ratio were also calculated.

### Measurement of lipoprotein profile

The concentrations of lipoprotein particle subclasses in the entire cohort were measured at baseline by LipoScience (Raleigh, NC, USA) with nuclear magnetic resonance spectroscopy using the LipoProfile-3 algorithm as described previously.^10, 11, 12, 13^ Lipoprotein particles were classified as HDL, LDL, intermediate-density lipoprotein (IDL) and very-LDL (VLDL) according to their diameters, with HDL subclassified as small, medium and large; LDL subclassified as small and large; and VLDL subclassified as small, medium and large (Supplementary Table S1). The mean lipoprotein particle sizes were the weighted average of the related subclasses. The CVs for the particle concentrations of VLDL (VLDL-P), LDL (LDL-P) and HDL (HDL-P) were all <4%. CVs for individual subclasses (large VLDL-P, medium VLDL-P, small VLDL-P, small LDL-P, large LDL-P, large HDL-P and small HDL-P) were <10%. CVs for IDL-P and medium HDL-P were 27.5%. CVs for the mean VLDL-P, LDL-P and HDL-P size were <2.0%.

### Other variables of interest

Information on age, race/ethnicity, education, marital status, smoking, alcohol use, physical activity, total gross family income and self-reported cancer were obtained using standardized questionnaires. The participant was asked to bring to the clinic containers for all medications used during the 2 weeks before the visit. The interviewer then recorded the name of each medication, the prescribed dose and frequency of administration from the containers. Physical activity was measured as the total number of hours of moderate and vigorous activity per week, multiplied by metabolic equivalent level.^14^

Participants wore light clothing and no shoes for measurement of height and weight. Body mass index (BMI) was measured as the weight in kilograms divided by height in meters squared. A standard flexible tape measure was used to measure hip and waist circumferences. Resting blood pressure was measured three times in a seated position with a Dinamap model Pro 100 automated oscillometric sphygmomanometer (Critikon, Tampa, FL, USA). The average of the last two blood pressure readings was used in the analysis.

Hypertension was defined as blood pressure ⩾140/90 mm Hg or use of antihypertensive medications. Diabetes was defined as fasting glucose ⩾126 mg dl^−1^ or use of glucose-lowering medications. C-reactive protein (CRP) was measured by immunonephelometry using a BNII nephelometer (N High Sensitivity CRP; Dade Behring, Deerfield, IL, USA). The assay range is 0.175–1100 mg l^−1^. Intra-assay CVs range from 2.3 to 4.4% and inter-assay CVs range from 2.1 to 5.7%. Fibrinogen antigen was measured using the BNII nephelometer (N Antiserum to Human Fibrinogen; Dade Behring). The intra-assay and inter-assay CVs are 2.7% and 2.6%, respectively. Interleukin-6 (IL-6) was measured using ultrasensitive enzyme-linked immunosorbent assay (Quantikine HS Human IL-6 Immunoassay; R&D Systems, Minneapolis, MN, USA) with a CV of 6.3% and a detection range of 0.156–10.0 pg ml^−1^. Estimated glomerular filtration rate was calculated using the creatinine-based Chronic Kidney Disease Epidemiology Collaboration equation.^15^ Serum creatinine was measured by rate reflectance spectrophotometry using thin film adaptation of the creatine amidinohydrolase method on the Vitros analyzer (Johnson & Johnson Clinical Diagnostics, Rochester, NY, USA) with a CV of 2.2%. The reference range in adult females was 0.4–1.1 mg dl^−1^ and in adult males was 0.5–1.2 mg dl^−1^.

### Statistical analysis

Data analysis was performed using SPSS 22 (IBM, Armonk, NY, USA) and STATA 14.0 (StataCorp, College Station, TX, USA). Data were presented as mean (s.d.) or percentage (number). For variables with a skewed distribution, data were presented as the median (interquartile range) and log-transformed before analysis. Comparison of baseline clinical characteristics between two groups of participants was performed by independent *t*-test for continuous variables and *χ*^2^-test for categorical variables, respectively. Those variables with a *P*<0.2 were used as covariates in subsequent regression analysis. As lipid-lowering medications (statins, fibrates, niacin and/or bile-acid sequestrants) can affect lipid and lipoprotein concentrations, lipid-lowering medication was adjusted for as a covariate in all subsequent regression analyses.

The associations of conventional measures of lipids or lipoprotein distribution (all as continuous variables) with the development of EDS at follow-up events were assessed using Cox proportional hazard regression analysis after adjustment for confounding factors. In this analysis, for each participant who developed EDS, the time to event (having EDS) was considered as the time interval between the date of the visit at which EDS was ascertained and the date of baseline visit 1. For participants who remained event-free, the follow-up time was censored at their last available visit. The proportional hazards assumption was checked using Schoenfeld residuals; we found violations for age and gender. Subsequent exploratory analysis led to inclusion of age-squared in the model, and treating the gender association as time-dependent (that is, the hazards ratio (HR) for gender changes as a function of time). *P* for interaction was estimated by including the multiplicative interaction term in the regression models in the full sample after adjustment for the main effects of the covariates. Multivariable linear regression analysis using robust s.e. estimation was used to assess the association of plasma lipids at baseline with absolute change in CES-D score between visits 1 and 5 among patients without EDS at baseline and self-reported use of antidepressant medications at visit 5. In all regression analyses, replacement of BMI by waist-to-hip ratio and height in the adjustment model made little difference to the results (data not shown). No multicollinearity was detected (variance inflation factors<4.0 in all the analyses). A two-tailed *P*<0.05 was considered statistically significant.

## Results

### Participant characteristics

The characteristics of the 4938 MESA-included participants and the 1876 excluded participants are shown in Supplementary Table S2. Sixty-eight percent of the excluded participants had EDS at baseline and 27% took antidepressant medications. Compared with these excluded participants, the 4938 participants included in the analysis were more likely to be male, more educated, married, physically active and less obese, and with lower family income, prevalence of diabetes and hypertension, circulating levels of inflammatory markers. They were also less likely to be Hispanic American or current smokers with less pack years of smoking.

Of the 4938 MESA participants without EDS at baseline, 1178 (23.9%) developed EDS over a mean follow-up period of 7.4 years. As shown in Table 1, participants who developed EDS were more likely to be younger, female, Caucasian or Hispanic American, less educated and current smokers with higher pack years of smoking, lower family income, higher BMI, higher estimated glomerular filtration rate and less likely to be married than those participants who did not develop EDS.

### Association of lipid and lipoprotein profile with incident EDS

Table 2 shows the lipid profile of participants with and without development of EDS during the follow-up period among 4938 participants without EDS at baseline. Participants with incident EDS had slightly, but statistically significantly, lower concentrations of total, LDL and non-HDL cholesterol, and lower atherogenic indices (total/HDL cholesterol ratio and LDL/HDL cholesterol ratio) than those who did not develop EDS. Participants with incident EDS also had a slightly lower concentration of IDL-P.

In multivariable Cox regression analysis, lower concentrations of total, LDL and non-HDL cholesterol, as well as lower atherogenic indices, were significantly associated with a higher risk of incident EDS (HRs=1.10–1.12 per s.d. decrease; model 3, Table 3). Among different lipoprotein subclass concentrations and sizes, a lower concentration of large HDL-P was significantly associated with a lower risk of incident EDS (model 3, Table 3). The association of lower concentrations of total cholesterol (HR (95% confidence interval (CI))=1.11 (1.03–1.19) per s.d. decrease, *P*=0.004), LDL cholesterol (HR (95% CI)=1.10 (1.03–1.17) per s.d. decrease, *P*=0.004), non-HDL cholesterol (HR (95% CI)=1.11 (1.03–1.19) per s.d. decrease, *P*=0.004), total/HDL cholesterol ratio (HR (95% CI)=1.11 (1.00–1.22) per s.d. decrease, *P*=0.04) and LDL/HDL cholesterol ratio (HR (95% CI)=1.01 (1.01–1.19) per s.d. decrease, *P*=0.02) with a higher incident EDS risk remained significant after further adjustment for large HDL-P concentration. However, the associations of large HDL-P concentration with incident EDS risk were not significant after further adjustment for total/HDL cholesterol ratio or LDL/HDL cholesterol ratio (*P*=1.00 and 0.96, respectively). Similar results were obtained after excluding 786 (15.9%) participants taking any lipid-lowering medication at baseline (data not shown).

### Sensitivity analysis using alternative definition of incident EDS

In a sensitivity analysis, EDS was defined as a CES-D score of ⩾21 or self-reported use of antidepressant medications. Using this definition, a total of 5236 participants without EDS at baseline were included in the analysis using the same subject exclusion criteria as before. Of these, 909 (17.4%) developed EDS during the follow-up period. As shown in Supplementary Table S3, lower concentrations of total, LDL and non-HDL cholesterol, and lower ratios of total/HDL cholesterol and LDL/HDL cholesterol at baseline, were significantly associated with a higher risk of incident EDS after adjustment for confounding factors (all *P*<0.01). No significant association was found for HDL cholesterol and triglyceride concentrations or for the concentration and size of different lipoprotein particle subclasses.

In a separate analysis, EDS was defined as a CES-D score of ⩾16 only, regardless of whether the participants took antidepressant medications. Using this definition, a total of 5274 participants without EDS at baseline were included in the analysis using the same subject exclusion criteria as before. Of these, 991 (18.8%) developed EDS during the follow-up period. As shown in Supplementary Table S4, lower concentrations of total, LDL and non-HDL cholesterol were associated with a higher risk of incident EDS (all *P*<0.05). No significant association was found for other lipid measures and concentrations and size of different lipoprotein particle subclasses.

### Subgroup analysis

As total, LDL and non-HDL cholesterol concentrations showed significant association with incident EDS in the sensitivity analysis, subsequent analysis focused on these three lipid measures. In subgroup analysis, the associations of lower concentrations of total, LDL and non-HDL cholesterol with higher incident EDS risk (defined as CES-D score of ⩾21 or self-reported use of antidepressant medications) did not differ significantly across different subgroups of age, sex, race/ethnicity, BMI, conventional lipid measures, atherogenic indices and CES-D score at baseline (all *P*-values for interactions >0.05, Supplementary Table S5).

### Association of conventional lipid measures with absolute change in CES-D score between exams 1 and 5

Of the 4938 participants without EDS at baseline, 3485 had valid data on CES-D score in the absence of antidepressant medication at both visits 1 and 5. Their mean CES-D score increased by 1.6 (s.d. 6.3) from 5.1 (s.d. 4.1) at visit 1 to 6.7 (s.d. 6.4) at visit 5. As shown in Table 4, lower concentrations of total, LDL and non-HDL cholesterol at baseline were significantly associated with an increase in CES-D score during the follow-up period. No significant interaction with race/ethnicity and sex was found.

### Association of changes in conventional lipid measures with subsequent risk of incident EDS

There were 3919 participants with valid data on lipid profile who did not have EDS (CES-D score of ⩾16 or self-reported use of antidepressant medications) at both baseline visit 1 and visit 3 (a mean period of 3.2 years), and had valid data on incident EDS after visit 3. Among these 3919 participants, 576 people (14.7%) developed incident EDS over a mean follow-up period of 6.3 years after visit 3. As shown in Table 5, a smaller absolute or relative increase in total cholesterol and non-HDL cholesterol concentrations between baseline visit 1 and visit 3 was associated with a lower risk of incident EDS after visit 3 (HRs=0.88–0.90 per s.d. decrease, all *P*<0.05). No significant interaction was found with age, sex, race/ethnicity, BMI, conventional lipid measures and CES-D score at baseline (data not shown). Similar results were found when analysis was only performed among a subgroup of 2788 participants who did not take any lipid-lowering medication at both visits 1 and 3 (data not shown) or among a subgroup of 2824 participants not being treated with a statin at visits 1, 2 and/or 3 (Supplementary Table S6).

## Discussion

In this study, and over 9.5 years of follow-up, lower total, LDL and non-HDL cholesterol concentrations at baseline were found to be associated with higher risk for incident EDS, but not lipoprotein subclasses or particle size. The association of lower concentrations of total, LDL and non-HDL cholesterol at baseline with incident EDS did not differ significantly across subgroups of age, sex, race/ethnicity, BMI, baseline conventional measures of lipids and CES-D score. Similar results were obtained when using a higher cutoff point for the CES-D score (⩾21) to define EDS, when using CES-D score (⩾16) only to define EDS, or when analyzing the changes in CES-D score between baseline and last visit.

Some previous studies have reported a lower concentration of total and LDL cholesterol in patients with depressive symptoms^16, 17, 18, 19^ and incident depression.^1, 2^ The relationship between metabolic syndrome and markers such as serum cholesterol and mood is complex. For both disorders, impaired serotonergic neurotransmission and inflammatory factors have been suggested to be involved. Cholesterol has an important role in the central nervous system, and it has been suggested that a lower serum total cholesterol concentration may be associated with a decrease in cell membrane cholesterol content.^20^ There is evidence that membrane cholesterol can modulate activity of the serotonin transporter, SERT,^21^ which controls the extracellular concentrations of serotonin, and that altered brain serotonin uptake provides a plausible link between cholesterol and changes in mood.^22^ There are some data supporting this, with a trend for a negative correlation between auditory processing (linked to serotoninergic neurotransmission) and serum LDL in patients with major depressive disorder.^23^ Interestingly, rats treated with the lipid-lowering medication, simvastatin, had evidence of reduced SERT activity, based on platelet serotonin uptake, and reduced membrane microviscosity, in addition to some behavioral changes, relative to vehicle-treated control rats.^24^ It has also been hypothesized that a low plasma cholesterol level may also reduce the synthesis of neurosteroids,^25^ which are potent and effective neuromodulators, leading to stress and other psychiatric disorders such as anxiety, depression and aggressive mood disorders.^26^

The association of lower total cholesterol and depressive symptoms is more consistently reported in elderly people.^16, 17^ In a recent study of young adolescents, a higher concentration of total cholesterol was indeed found to be associated with a higher EDS risk.^27^ In the present study, data were adjusted for age, and subgroup analysis did not reveal a significant interaction with age. Therefore, our study did not provide evidence that age is an important effect-modifying factor, although our study comprised middle and older age participants. For HDL cholesterol, a recent study has reported the association of a lower plasma HDL cholesterol concentration with a higher risk of depression in elderly men.^2^ However, we did not find any significant association of HDL cholesterol concentration with incident EDS risk in the present study. The discrepancy could be due to differences in subject characteristics such as age, education level, use of lipid-lowering medications and smoking status, as well as the use of different variables being adjusted in the regression models, and different tests for assessment of depressive symptoms.

Despite being associated with lower CVD risk, lower plasma concentrations of total, LDL and non-HDL cholesterol at baseline are associated with a modestly higher incident EDS risk in the present study. On the other hand, depression has also been suggested as a risk factor for CVD.^28, 29^ This paradoxical relationship could be confounded by other factors related to depression and CVD. Possible confounding factors are systemic inflammation,^30^ and the associated pro-atherogenic lipoprotein profile.^31^ In a previous small study of 65 depressed patients and 33 healthy controls, the depressed patients had lower total and LDL cholesterol concentrations, and higher levels of small, dense LDL particles than the healthy controls, which may explain the paradoxical relationship of low cholesterol concentration and high CVD risk in depression.^18^ However, in the present study with a much larger sample size, we did not find a significant independent association between atherogenic indices and lipoprotein profiles using nuclear magnetic resonance spectroscopy with incident EDS risk. We did not find a significant difference in CRP and IL-6 levels between participants with and without incident EDS. The association between lipids and incident EDS risk remained significant in the full adjustment model (including IL-6 as covariate). Therefore, our study does not support an important role of inflammation in the modest association between lipids and incident EDS. There is evidence of sex-specific relationships in the literature. For instance, recent work linked atherogenic indices with a faster rate of increase in depressive symptoms in women only.^32^ In another Korean study of 15 073 men and 15 034 women, depressive symptoms were associated with the metabolic syndrome in women only, but were inversely associated with the hypertension component of the metabolic syndrome in men.^33^ A similar trend was found in our study, in which the association of lower total cholesterol concentration with EDS risk was found only in men (HR=1.15, *P*=0.009), but not in women (HR=1.09, *P*=0.07), although the sex interaction did not reach statistical significance (Supplementary Table S5).

Although a lower baseline total cholesterol concentration was found to be associated with higher incident EDS over the whole follow-up period in the present study, an interesting and contradictory result was found when we analyzed the relationship of changes in lipid levels from baseline to visit 3 with subsequent risk of incident EDS. In this analysis, a larger increase in total cholesterol concentration was associated with a higher subsequent risk. We are unclear for the discrepancy in the findings between baseline lipid levels and the changes in lipid levels over initial 3.2 years of follow-up. Nevertheless, our study does not provide support for reducing the risk of EDS by increasing total cholesterol concentrations.

In a recent study of 24 216 postmenopausal women, the association of lower LDL cholesterol with incident EDS was only found in those without lipid-lowering therapy, but not in those with lipid-lowering therapy.^34^ In this regard, statins are commonly used to lower the concentration of total and LDL cholesterol. However, we did not find any significant interaction with lipid-lowering therapy or use of statin in the association of lower total, LDL and non-HDL cholesterol concentrations with higher EDS risk (data not shown). In fact, the relationship of the use of statins with depression is inconsistent, with some studies showing that statins increase depression risk,^25, 35^ and others having the opposite effect.^36, 37, 38^ Further investigations are needed to elucidate this paradoxical relationship between total cholesterol concentration and EDS.

Our study has the advantage of making use of data with a longitudinal study design and good quality control of a large well-characterized sample of clinically apparently healthy participants. However, there are several limitations in our study. In our analysis, participants were defined as having EDS if they reported the use of antidepressant medications. However, these medications can also be used for other conditions such as anxiety, eating disorders, smoking cessation, insomnia, post-herpetic pain and migraines, and the reasons for the use of antidepressant medications were not recorded in the MESA study. This may lead to the apparently high incident EDS rate. In a retrospective analysis of Georgia Medicaid recipients, ~75% antidepressant recipients reported off-label use of antidepressant medications.^39^ However, in the present study, similar findings were obtained in a separate analysis when EDS was defined using the CES-D score, without the use of data on antidepressant medications. Moreover, the absolute differences in blood lipid concentrations between participants with and without incident EDS were modest and clinical interpretation should be cautious. Our study may also suffer from false-positive findings due to multiple comparisons. However, as different parameters of conventional lipid measures, and lipoprotein particle concentrations and sizes were highly correlated with each other, multiple testing correction was not performed.

In conclusion, although lower baseline concentrations of total cholesterol, LDL cholesterol and non-HDL cholesterol are modestly associated with incident EDS, here a larger increase in total cholesterol also predicted a modestly higher subsequent risk of EDS. Further studies are needed to investigate whether our findings can be found in other cohorts with younger participants, and the underlying mechanisms of the contradictory relationship between cholesterol concentrations and EDS. Nevertheless, the beneficial cardiovascular effects of lipid-lowering by drugs such as statins outweigh the potential small and modest effects on EDS. Our study does not support increasing cholesterol levels to reduce EDS.

## Figures and Tables

**Table 1:** Baseline clinical characteristics of participants with and without EDS developed during follow-up

*Characteristics*	*Without EDS*	*With EDS*	P
*N*	3760	1178	—
Age, years	62.3 (10.0)	61.2 (10.2)	0.001
Women, %	46.4 (1744)	58.1 (685)	<0.001

*Race/ethnicity, %*			
Caucasian	38.3 (1440)	41.2 (485)	<0.001
African American	29.5 (1108)	24.7 (291)	
Hispanic American	18.6 (698)	23.6 (278)	
Chinese American	13.7 (514)	10.5 (124)	

*Education, %*			
<High school	14.2 (533)	19.4 (228)	<0.001
High school	41.1 (1543)	43.3 (510)	
>High school	44.7 (1680)	37.3 (439)	

*Smoking, %*			
Never	51.9 (1949)	48.2 (567)	<0.001
Former	37.2 (1399)	36.8 (433)	
Current	10.9 (409)	15.0 (177)	
Pack years of smoking	10.4 (19.7)	12.2 (23.0)	0.01
Current alcohol use, %	57.6 (2155)	57.5 (675)	0.95

*Total gross family income*			
<$30 000	31.7 (1150)	38.5 (439)	<0.001
$30 000–$74 999	41.1 (1491)	40.2 (458)	
⩾$75 000	27.2 (985)	21.3 (243)	

*Marital status, %*			
Married	66.0 (2464)	60.4 (704)	<0.001
Widowed/divorced/separated	26.8 (1001)	30.0 (350)	
Single	7.2 (268)	9.5 (111)	
Physical activity, MET-hours per weeks	98 (98)	97 (95)	0.72
BMI, kg/m^2^	28.0 (5.2)	28.5 (5.6)	0.02
Waist-to-hip ratio	0.927 (0.080)	0.924 (0.080)	0.17
Heart rate, beats per minute	62.6 (9.4)	63.1 (9.6)	0.08
Diabetes, %	10.9 (409)	12.1 (142)	0.27
Hypertension, %	42.8 (1610)	45.0 (530)	0.19
Self-reported cancer, %	8.0 (302)	6.7 (79)	0.13
Any lipid-lowering medication, %	15.8 (595)	16.2 (191)	0.75
CRP, mg l^−1^ a	1.79 (0.80–4.02)	1.90 (0.84–4.12)	0.53
Fibrinogen, mg dl^−1^	344 (72)	344 (72)	0.87
IL-6, pg ml^−1^ a	1.15 (0.75–1.83)	1.14 (0.75–1.73)	0.16
eGFR, ml min^−^^1^ 1.73 m^−2^	77.6 (15.9)	79.3 (16.0)	0.002

Abbreviations: BMI, body mass index; CRP, C-reactive protein; EDS, elevated depressive symptoms; eGFR, estimated glomerular filtration rate; IL-6, interleukin-6; MET, metabolic equivalent.

Data are expressed as mean (s.d.), percent (*n*) or median (interquartile range), where appropriate. *P-*values were estimated by *t*-test for continuous variables and *χ*^2^-test for categorical variables, respectively.

a *P-*values were estimated using ln-transformed data.

**Table 2:** Baseline lipid profile of participants with and without EDS developed during follow-up

*Lipid and lipoprotein profile*	*Without EDS*	*With EDS*	P
*Conventional lipid measure*			
Total cholesterol, mg dl^−1^	193.5 (34.3)	192.8 (34.1)	0.02
LDL cholesterol, mg dl^−1^	118.2 (30.9)	115.7 (30.8)	0.003
HDL cholesterol, mg dl^−1^^a^	48 (40–58)	49 (41–60)	0.52
Non-HDL cholesterol, mg dl^−1^	143.0 (34.0)	140.9 (34.2)	0.01
Triglycerides, mg dl^−1^^a^	108 (77–155)	111 (77–160)	0.79
Total/HDL cholesterol ratio	4.08 (1.17)	3.96 (1.15)	0.04
LDL/HDL cholesterol ratio	2.52 (0.93)	2.41 (0.91)	0.01

*Lipoprotein particle concentration*			
*VLDL-P, nmol l^−1^*			
Total^a^	62.1 (39.9–87.9)	63.2 (40.2–88.4)	0.58
Large^a^	2.4 (0.8–6.1)	2.6 (0.9–6.5)	0.70
Medium^a^	23.4 (12.1–40.6)	23.8 (11.9–39.5)	0.60
Small^a^	31.5 (19.6–44.8)	32.7 (20.4–45.4)	0.74
			
IDL-P, nmol l^−1^^a^	102 (50–176)	103 (51–174)	0.04
			
* LDL-P, nmol l*^−1^			
Total	1131 (304)	1110 (317)	0.06
Large	584 (252)	602 (259)	0.89
Small	547 (374)	508 (375)	0.09
			
* HDL-P, μmol l*^−*1*^			
Total	33.7 (6.5)	34.6 (6.7)	0.18
Large^a^	5.0 (3.3–7.6)	5.3 (3.6–8.2)	0.14
Medium^a^	12.2 (8.3–16.6)	12.8 (8.9–17.5)	0.62
Small	14.9 (5.5)	14.6 (5.8)	0.87

*Lipoprotein particle size, nm*			
VLDL-P	48.2 (7.7)	48.5 (7.8)	0.93
LDL-P	20.7 (0.5)	20.8 (0.5)	0.75
HDL-P	9.2 (0.5)	9.3 (0.5)	0.10

Abbreviations: EDS, elevated depressive symptoms; HDL, high-density lipoprotein; HDL-P, high-density lipoprotein particle; IDL-P, intermediate-density lipoprotein particle; LDL, low-density lipoprotein; LDL-P, low-density lipoprotein particle; VLDL-P, very-low-density lipoprotein particle.

Data are expressed as mean (s.d.) or median (interquartile range). *P-*values were estimated by multivariable linear regression model with continuous measures of lipid and lipoprotein profile as the dependent variable after adjusting for age, sex and race/ethnicity.

a *P*-values were estimated using ln-transformed data.

**Table 3:** Associations of lower lipid and lipoprotein profiles at baseline with incident EDS

*Lipid and lipoprotein profile*	*s.d.*	*Model 1*		*Model 2*		*Model 3*	
		*HR (95% CI)*	P	*HR (95% CI)*	P	*HR (95% CI)*	P
*Conventional lipid measure*							
Total cholesterol, mg d1^−1^	34.2	1.08 (1.02–1.15)	0.008	1.10 (1.03–1.17)	0.003	1.12 (1.05–1.20)	0.001
LDL cholesterol, mg d1^−1^	30.9	1.10 (1.03–1.16)	0.002	1.11 (1.04–1.18)	0.001	1.11 (1.04–1.18)	0.001
HDL cholesterol, mg d1^−1^	14.7	1.01 (0.95–1.07)	0.78	0.99 (0.92–1.06)	0.77	1.00 (0.93–1.08)	0.97
Non-HDL cholesterol, mg d1^−1^	34.1	1.08 (1.02–1.14)	0.01	1.10 (1.04–1.17)	0.002	1.12 (1.05–1.20)	0.001
Triglycerides, mg d1^−1^	64.6	0.98 (0.92–1.03)	0.41	1.01 (0.95–1.08)	0.79	1.00 (0.94–1.07)	0.94
Total/HDL cholesterol ratio	1.17	1.05 (0.98–1.11)	0.16	1.08 (1.01–1.15)	0.03	1.11 (1.02–1.20)	0.01
LDL/HDL cholesterol ratio	0.93	1.06 (1.00–1.13)	0.05	1.09 (1.02–1.17)	0.01	1.10 (1.02–1.18)	0.01

*Lipoprotein particle concentration*							
*VLDL-P, nmol l^−1^*							
Total	35.2	0.98 (0.93–1.05)	0.61	1.02 (0.95–1.08)	0.64	0.98 (0.89–1.07)	0.66
Large	5.4	0.98 (0.92–1.04)	0.49	1.01 (0.95–1.08)	0.69	1.07 (0.94–1.21)	0.30
Medium	21.3	0.98 (0.92–1.04)	0.56	1.00 (0.94–1.07)	0.90	0.99 (0.90–1.08)	0.76
Small	19.4	1.00 (0.94–1.06)	0.91	1.02 (0.96–1.08)	0.56	0.98 (0.92–1.05)	0.62
IDL-P, nmol l^−1^	94.9	1.04 (0.98–1.11)	0.16	1.05 (0.99–1.12)	0.10	1.01 (0.93–1.09)	0.85
*LDL-P, nmol l*^−*1*^							
Total	308	1.05 (0.99–1.12)	0.09	1.07 (1.01–1.14)	0.03	0.99 (0.91–1.09)	0.88
Large	254	1.01 (0.95–1.08)	0.67	1.01 (0.94–1.07)	0.87	0.93 (0.85–1.02)	0.11
Small	375	1.04 (0.98–1.10)	0.24	1.06 (0.99–1.13)	0.08	1.05 (0.95–1.15)	0.33
*HDL-P, μmol l^−1^*							
Total	6.6	0.96 (0.90–1.02)	0.22	0.96 (0.90–1.03)	0.28	0.96 (0.87–1.06)	0.40
Large	3.4	0.97 (0.92–1.03)	0.39	0.95 (0.89–1.02)	0.14	0.86 (0.74–0.99)	0.04
Medium	6.8	1.00 (0.94–1.06)	0.95	1.00 (0.94–1.07)	0.98	1.01 (0.94–1.08)	0.75
Small	5.6	0.98 (0.92–1.04)	0.46	0.99 (0.93–1.05)	0.74	0.98 (0.92–1.05)	0.62
*Lipoprotein particle size, nm*							
VLDL-P	7.8	0.98 (0.92–1.04)	0.54	1.01 (0.95–1.08)	0.68	1.06 (0.96–1.16)	0.20
LDL-P	0.5	1.01 (0.95–1.07)	0.75	0.99 (0.93–1.06)	0.76	0.98 (0.89–1.07)	0.62
HDL-P	0.5	0.96 (0.90–1.02)	0.20	0.93 (0.87–1.00)	0.05	0.92 (0.84–1.01)	0.07

Abbreviations: CI, confidence interval; EDS, elevated depressive symptoms; HDL, high-density lipoprotein; HDL-P, high-density lipoprotein particle; HR, hazards ratio; IDL-P, intermediate-density lipoprotein particle; LDL, low-density lipoprotein; LDL-P, low-density lipoprotein particle; VLDL-P, very-low-density lipoprotein particle.

HR is expressed in terms of per s.d. decrease in each lipid measure.

Model 1: adjusted for age, age-squared, sex (as both time-independent and -dependent variables) and race/ethnicity.

Model 2: Further adjusted for education, smoking, pack years of smoking, total gross family income, marital status, any lipid-lowering medication (yes or no), body mass index, heart rate, hypertension, self-reported cancer, interleukin-6 and estimated glomerular filtration rate.

Model 3: Further adjusted for HDL cholesterol (except for total/HDL cholesterol ratio and LDL/HDL cholesterol ratio), LDL cholesterol (except for total cholesterol, non-HDL cholesterol, total/HDL cholesterol ratio and LDL/HDL cholesterol ratio) and triglycerides, where appropriate.

**Table 4:** Associations of lower levels of conventional lipid measures at baseline with absolute change in CES-D score during follow-up

*Conventional lipid measures*	β	P	P *for interaction with race/ethnicity*	P *for interaction with sex*
Total cholesterol	0.051	0.02	0.34	0.53
LDL cholesterol	0.043	0.02	0.61	0.74
HDL cholesterol^a^	0.002	0.94	0.17	0.45
Non-HDL cholesterol	0.049	0.02	0.71	0.70
Triglycerides^a^	−0.004	0.86	0.65	0.85

Abbreviations: CES-D, Center for Epidemiological Studies Depression Scale; HDL, high-density lipoprotein; LDL, low-density lipoprotein.

Data are expressed as standardized regression coefficient (*β*) per s.d. decrease in each lipid measure in multivariable linear regression with absolute change in CES-D score as the dependent variable.

Model 1: Adjusted for baseline CES-D score, age, sex, race/ethnicity, education, smoking, pack years of smoking, total gross family income, marital status, body mass index, heart rate, hypertension, self-reported cancer, log-transformed interleukin-6, estimated glomerular filtration rate, log-transformed HDL cholesterol, LDL cholesterol (except total cholesterol and non-HDL cholesterol) and log-transformed triglycerides, and history of lipid-lowering medication usage at visits 1 and 5 (‘no use at both visits', ‘use at visit 1, but no use at visit 5', ‘no use at visit 1, but use at visit 5' and ‘use at both visits'), where appropriate.

a *P*-values were estimated using log-transformed data.

**Table 5:** Associations of smaller changes in conventional lipid measures with subsequent risk of incident EDS (*n*=3919)

*Conventional lipid measure*	*s.d.*	*HR (95% CI)*	P
*Absolute change, mg* *dl*^−*1*^			
Total cholesterol	31.6	0.89 (0.80–1.00)	0.048
LDL cholesterol	29.0	0.90 (0.81–1.00)	0.06
HDL cholesterol	8.2	1.02 (0.93–1.12)	0.69
Non-HDL cholesterol	31.5	0.89 (0.79–0.99)	0.04
Triglycerides	52.4	0.95 (0.86–1.04)	0.28

*Relative change, %*			
Total cholesterol	15.9	0.88 (0.80–0.99)	0.03
LDL cholesterol	25.8	0.92 (0.85–1.00)	0.054
HDL cholesterol	16.0	1.01 (0.92–1.10)	0.85
Non-HDL cholesterol	21.9	0.90 (0.81–0.99)	0.03
Triglycerides	43.0	0.92 (0.84–1.01)	0.08

Abbreviations: CI, confidence interval; EDS, elevated depressive symptoms; HDL, high-density lipoprotein; HR, hazards ratio; LDL, low-density lipoprotein.

HR is expressed in terms of per s.d. decrease in the change of each lipid measure from baseline visit 1 to visit 3.

Data were adjusted for age, age-squared, sex (as both time-independent and -dependent variables), race/ethnicity, education, smoking, pack years of smoking, total gross family income, marital status, body mass index, heart rate, hypertension, self-reported cancer, interleukin-6, estimated glomerular filtration rate, corresponding lipid levels at baseline visit 1, HDL cholesterol, LDL cholesterol (except for total cholesterol and non-HDL cholesterol) and triglycerides at visit 1, history of lipid-lowering medication usage at visits 1 and 3 (‘no use at both visits', ‘use at visit 1, but no use at visit 3', ‘no use at visit 1, but use at visit 3' and ‘use at both visits'), time between visit 1 and visit 3, and change in weight from visit 1 to visit 3.

## References

[bib1] Partonen T, Haukka J, Virtamo J, Taylor PR, Lönnqvist J. Association of low serum total cholesterol with major depression and suicide. Br J Psychiatry 1999; 175: 259–262. 10645328 10.1192/bjp.175.3.259

[bib2] Almeida OP, Yeap BB, Hankey GJ, Golledge J, Flicker L. HDL cholesterol and the risk of depression over 5 years. Mol Psychiatry 2014; 19: 637–638. 23999523 10.1038/mp.2013.113

[bib3] Bild DE, Bluemke DA, Burke GL, Detrano R, Diez Roux AV, Folsom AR et al. Multi-ethnic study of atherosclerosis: objectives and design. Am J Epidemiol 2002; 156: 871–881. 12397006 10.1093/aje/kwf113

[bib4] Radloff LS. The CES-D scale: a self-report depression scale for research in the general population. Appl Psychol Meas 1977; 1: 385–401.

[bib5] Beekman AT, Deeg DJ, Van Limbeek J, Braam AW, De Vries MZ, Van Tilburg W. Criterion validity of the Center for Epidemiologic Studies Depression scale (CES-D): results from a community-based sample of older subjects in The Netherlands. Psychol Med 1997; 27: 231–235. 9122304 10.1017/s0033291796003510

[bib6] Remigio-Baker RA, Allison MA, Schreiner PJ, Szklo M, Crum RM, Leoutsakos JM et al. Difference by sex but not by race/ethnicity in the visceral adipose tissue-depressive symptoms association: the Multi-Ethnic Study of Atherosclerosis. Psychoneuroendocrinology 2014; 47: 78–87. 25001957 10.1016/j.psyneuen.2014.05.004 PMC4134940

[bib7] Golden SH, Lazo M, Carnethon M, Bertoni AG, Schreiner PJ, Diez Roux AV et al. Examining a bidirectional association between depressive symptoms and diabetes. JAMA 2008; 299: 2751–2759. 18560002 10.1001/jama.299.23.2751 PMC2648841

[bib8] Zich JM, Attkisson CC, Greenfield TK. Screening for depression in primary care clinics: the CES-D and the BDI. Int J Psychiatry Med 1990; 20: 259–277. 2265888 10.2190/LYKR-7VHP-YJEM-MKM2

[bib9] Friedewald WT, Levy RI, Fredrickson DS. Estimation of the concentration of low-density lipoprotein cholesterol in plasma, without use of the preparative ultracentrifuge. Clin Chem 1972; 18: 499–502. 4337382

[bib10] Mora S, Szklo M, Otvos JD, Greenland P, Psaty BM, Goff DCJr et al. LDL particle subclasses, LDL particle size, and carotid atherosclerosis in the Multi-Ethnic Study of Atherosclerosis (MESA). Atherosclerosis 2007; 192: 211–217. 16765964 10.1016/j.atherosclerosis.2006.05.007

[bib11] de Boer IH, Astor BC, Kramer H, Palmas W, Rudser K, Seliger SL et al. Mild elevations of urine albumin excretion are associated with atherogenic lipoprotein abnormalities in the Multi-Ethnic Study of Atherosclerosis (MESA). Atherosclerosis 2008; 197: 407–414. 17681346 10.1016/j.atherosclerosis.2007.06.018 PMC2288670

[bib12] Mackey RH, Greenland P, Goff DCJr, Lloyd-Jones D, Sibley CT, Mora S. High-density lipoprotein cholesterol and particle concentrations, carotid atherosclerosis, and coronary events: MESA (multi-ethnic study of atherosclerosis). J Am Coll Cardiol 2012; 60: 508–516. 22796256 10.1016/j.jacc.2012.03.060 PMC3411890

[bib13] Ong KL, Ding J, McClelland RL, Cheung BM, Criqui MH, Barter PJ et al. Relationship of pericardial fat with lipoprotein distribution: the Multi-Ethnic study of atherosclerosis. Atherosclerosis 2015; 241: 664–670. 26117404 10.1016/j.atherosclerosis.2015.06.027 PMC4510019

[bib14] Bertoni AG, Whitt-Glover MC, Chung H, Le KY, Barr RG, Mahesh M et al. The association between physical activity and subclinical atherosclerosis: the Multi-Ethnic Study of Atherosclerosis. Am J Epidemiol 2009; 169: 444–454. 19075250 10.1093/aje/kwn350 PMC2726643

[bib15] Levey AS, Stevens LA, Schmid CH, Zhang YL, Castro AF3rd, Feldman HI et al. A new equation to estimate glomerular filtration rate. Ann Intern Med 2009; 150: 604–612. 19414839 10.7326/0003-4819-150-9-200905050-00006 PMC2763564

[bib16] Morgan RE, Palinkas LA, Barrett-Connor EL, Wingard DL. Plasma cholesterol and depressive symptoms in older men. Lancet 1993; 341: 75–79. 8093404 10.1016/0140-6736(93)92556-9

[bib17] Aijänseppä S, Kivinen P, Helkala EL, Kivelä SL, Tuomilehto J, Nissinen A. Serum cholesterol and depressive symptoms in elderly Finnish men. Int J Geriatr Psychiatry 2002; 17: 629–634. 12112160 10.1002/gps.666

[bib18] Hummel J, Westphal S, Weber-Hamann B, Gilles M, Lederbogen F, Angermeier T et al. Serum lipoproteins improve after successful pharmacologic antidepressant treatment: a randomized open-label prospective trial. J Clin Psychiatry 2011; 72: 885–991. 21294998 10.4088/JCP.09m05853blu

[bib19] Vilibić M, Jukić V, Pandžić-Sakoman M, Bilić P, Milošević M. Association between total serum cholesterol and depression, aggression, and suicidal ideations in war veterans with posttraumatic stress disorder: a cross-sectional study. Croat Med J 2014; 55: 520–529. 25358885 10.3325/cmj.2014.55.520 PMC4228297

[bib20] Engelberg H. Low serum cholesterol and suicide. Lancet 1992; 339: 727–729. 1347593 10.1016/0140-6736(92)90609-7

[bib21] Scanlon SM, Williams DC, Schloss P. Membrane cholesterol modulates serotonintransporter activity. Biochemistry 2001; 40: 10507–10513. 11523992 10.1021/bi010730z

[bib22] Owens MJ, Nemeroff CB. Role of serotonin in the pathophysiology of depression: focus on the serotonin transporter. Clin Chem 1994; 40: 288–295. 7508830

[bib23] Park YM, Lee BH, Lee SH. The association between serum lipid levels, suicide ideation, and central serotonergic activity in patients with major depressive disorder. J Affect Disord 2014; 159: 62–65. 24679391 10.1016/j.jad.2014.01.016

[bib24] Vevera J, Valeš K, Fišar Z, Hroudová J, Singh N, Stuchlík A et al. The effect of prolonged simvastatin application on serotonin uptake, membrane microviscosity and behavioral changes in the animal model. Physiol Behav 2016; 158: 112–120. 26917054 10.1016/j.physbeh.2016.02.029

[bib25] You H, Lu W, Zhao S, Hu Z, Zhang J. The relationship between statins and depression: a review of the literature. Expert Opin Pharmacother 2013; 14: 1467–1476. 23767773 10.1517/14656566.2013.803067

[bib26] Zorumski CF, Paul SM, Izumi Y, Covey DF, Mennerick S. Neurosteroids, stress and depression: potential therapeutic opportunities. Neurosci Biobehav Rev 2013; 37: 109–122. 23085210 10.1016/j.neubiorev.2012.10.005 PMC3591791

[bib27] Jeffery AN, Hyland ME, Hosking J, Wilkin TJ. Mood and its association with metabolic health in adolescents: a longitudinal study, EarlyBird 65. Pediatr Diabetes 2014; 15: 599–605. 24552539 10.1111/pedi.12125

[bib28] Bradley SM, Rumsfeld JS. Depression and cardiovascular disease. Trends Cardiovasc Med 2015; 25: 614–622. 25850976 10.1016/j.tcm.2015.02.002

[bib29] Cohen BE, Edmondson D, Kronish IM. State of the art review: depression, stress, anxiety, and cardiovascular disease. Am J Hypertens 2015; 28: 1295–1302. 25911639 10.1093/ajh/hpv047 PMC4612342

[bib30] Camacho Á, Larsen B, McClelland RL, Morgan C, Criqui MH, Cushman M et al. Association of subsyndromal and depressive symptoms with inflammatory markers among different ethnic groups: the multi-ethnic study of atherosclerosis (MESA). J Affect Disord 2014; 164: 165–170. 24856570 10.1016/j.jad.2014.04.018 PMC4079665

[bib31] Choy E, Sattar N. Interpreting lipid levels in the context of high-grade inflammatory states with a focus on rheumatoid arthritis: a challenge to conventional cardiovascular risk actions. Ann Rheum Dis 2009; 68: 460–469. 19286905 10.1136/ard.2008.101964

[bib32] Beydoun MA, Beydoun HA, Dore GA, Fanelli-Kuczmarski MT, Evans MK, Zonderman AB. Total serum cholesterol, atherogenic indices and their longitudinal association with depressive symptoms among US adults. Transl Psychiatry 2015; 5: e518. 25734511 10.1038/tp.2015.4 PMC4354360

[bib33] Rhee SJ, Kim EY, Kim SH, Lee HJ, Kim B, Ha K et al. Subjective depressive symptoms and metabolic syndrome among the general population. Prog Neuropsychopharmacol Biol Psychiatry 2014; 54: 223–230. 24975752 10.1016/j.pnpbp.2014.06.006

[bib34] Persons JE, Robinson JG, Coryell WH, Payne ME, Fiedorowicz JG. Longitudinal study of low serum LDL cholesterol and depressive symptom onset in postmenopause. J Clin Psychiatry 2016; 77: 212–220. 26930520 10.4088/JCP.14m09505 PMC4906804

[bib35] Kang JH, Kao LT, Lin HC, Tsai MC, Chung SD. Statin use increases the risk of depressive disorder in stroke patients: a population-based study. J Neurol Sci 2015; 348: 89–93. 25483831 10.1016/j.jns.2014.11.013

[bib36] Redlich C, Berk M, Williams LJ, Sundquist J, Sundquist K, Li X. Statin use and risk of depression: a Swedish national cohort study. BMC Psychiatry 2014; 14: 348. 25471121 10.1186/s12888-014-0348-y PMC4266881

[bib37] Young-Xu Y, Chan KA, Liao JK, Ravid S, Blatt CM. Long-term statin use and psychological well-being. J Am Coll Cardiol 2003; 42: 690–697. 12932603 10.1016/S0735-1097(03)00785-X PMC2673913

[bib38] Stafford L, Berk M. The use of statins after a cardiac intervention is associated with reduced risk of subsequent depression: proof of concept for the inflammatory and oxidative hypotheses of depression? J Clin Psychiatry 2011; 72: 1229–1235. 21208591 10.4088/JCP.09m05825blu

[bib39] Chen H, Reeves JH, Fincham JE, Kennedy WK, Dorfman JH, Martin BC. Off-label use of antidepressant, anticonvulsant, and antipsychotic medications among Georgia medicaid enrollees in 2001. J Clin Psychiatry 2006; 67: 972–982. 16848658 10.4088/jcp.v67n0615

