# Supplementary material for: Lipids, lipoprotein distribution and depressive symptoms: the Multi-Ethnic Study of Atherosclerosis
Source: Transl Psychiatry. 2016 Nov 29;6(11):e962–. doi: 10.1038/tp.2016.232 (PMC5290355; doi:10.1038/tp.2016.232)
Supplement: Supplementary Information [file tp2016232x1.doc]

**Supplementary Table S1.** Classification of lipoprotein particles by diameter

| **Subclass** | **Particle diameter, nm** |
| --- | --- |
| VLDL-P |  |
| Large | >60 |
| Medium | 35-60 |
| Small | 29-35 |
| IDL-P | 23-29 |
| LDL-P |  |
| Large | 20.5-23 |
| Small | 18-20.5 |
| HDL-P |  |
| Large | 9.4-14 |
| Medium | 8.2-9.4 |
| Small | 7.3-8.2 |

Abbreviations: HDL-P, high-density lipoprotein particle; IDL-P, intermediate-density lipoprotein particle; LDL-P, low-density lipoprotein particle; VLDL-P, very-low-density lipoprotein particle.

**Supplementary Table S2.** Baseline clinical characteristics between participants included and excluded in this study

| **Characteristics** | **Excluded** | **Included** | ***P*** |
| --- | --- | --- | --- |
| N | 1876 | 4938 | - |
| Age, years | 62.4 (10.7) | 62.0 (10.0) | 0.16 |
| Women, % | 62.5 (1,172) | 49.2 (2,429) | <0.001 |
| Race/ethnicity, % |  |  |  |
| Caucasian | 37.2 (697) | 39.0 (1925) | <0.001 |
| African American | 26.3 (493) | 28.3 (1399) |  |
| Hispanic American | 27.7 (520) | 19.8 (976) |  |
| Chinese American | 8.8 (166) | 12.9 (638) |  |
| Education, % |  |  |  |
| <High school | 25.0 (464) | 15.4 (761) | <0.001 |
| High school | 42.0 (780) | 41.6 (2053) |  |
| >High school | 33.0 (614) | 43.0 (2119) |  |
| Smoking, % |  |  |  |
| Never | 48.5 (902) | 51.0 (2516) | <0.001 |
| Former | 35.3 (655) | 37.1 (1832) |  |
| Current | 16.2 (301) | 11.9 (586) |  |
| Pack-years of smoking | 12.7 (21.8) | 10.8 (20.5) | 0.001 |
| Current alcohol use, % | 49.7 (919) | 57.6 (2830) | <0.001 |
| Total gross family income |  |  |  |
| <$30 000 | 48.6 (862) | 33.3 (1589) | <0.001 |
| $30 000- $74 999 | 37.4 (663) | 40.9 (1949) |  |
| ≥$75 000 | 14.1 (250) | 25.8 (1228) |  |
| Marital status, % |  |  |  |
| Married | 51.6 (951) | 64.7 (3168) | <0.001 |
| Widowed / divorced / separated | 38.7 (712) | 27.6 (1351) |  |
| Single | 9.7 (179) | 7.7 (379) |  |
| Physical activity, MET-hours/weeks | 90.4 (99.7) | 97.8 (97.7) | 0.006 |
| BMI, kg m^-2^ | 28.9 (5.9) | 28.1 (5.3) | <0.001 |
| Waist-to-hip ratio | 0.933 (0.081) | 0.926 (0.080) | 0.001 |
| Heart rate, beats per minute | 64.2 (10.2) | 62.7 (9.4) | <0.001 |
| Diabetes, % | 16.6 (308) | 11.2 (551) | <0.001 |
| Hypertension, % | 48.9 (918) | 43.3 (2140) | <0.001 |
| Self-reported cancer, % | 8.3 (155) | 7.7 (381) | 0.44 |
| Total cholesterol, mg d1^-1^ | 196.4 (39.4) | 193.3 (34.2) | 0.003 |
| LDL cholesterol, mg d1^-1^ | 116.1 (33.0) | 117.6 (30.9) | 0.081 |
| HDL cholesterol, mg d1^-1a^ | 49 (40-60) | 48 (40-59) | 0.38 |
| Non-HDL cholesterol, mg d1^-1^ | 145.0 (40.6) | 142.5 (34.1) | 0.02 |
| Total/HDL cholesterol ratio | 4.13 (1.45) | 4.05 (1.17) | 0.05 |
| LDL/HDL cholesterol ratio | 2.41 (0.97) | 2.49 (0.93) | 0.002 |
| Triglycerides, mg d1^-1a^ | 118 (81-173) | 109 (77-156) | <0.001 |
| Any lipid-lowering medication, % | 17.1 (319) | 15.9 (786) | 0.22 |
| CRP, mg l^-1a^ | 2.23 (0.90-4.87) | 1.81 (0.81-4.05) | <0.001 |
| Fibrinogen, mg d1^-1^ | 354 (78) | 344 (72) | <0.001 |
| IL-6, pg ml^-1a^ | 1.37 (0.89-2.15) | 1.15 (0.75-1.81) | <0.001 |
| eGFR, ml min^-1^ 1.73 m^-2^ | 78.1 (17.4) | 78.0 (15.9) | 0.85 |
| EDS, % | 68.0 (1245) | 0 (0) | <0.001 |
| Use of antidepressant medications, % | 27.0 (503) | 0 (0) | <0.001 |

Abbreviations: BMI, body mass index; CRP, C-reactive protein; EDS, elevated depressive symptoms; eGFR, estimated glomerular filtration rate; HDL, high-density lipoprotein; IL-6, interleukin-6; LDL, low-density lipoprotein; MET, metabolic equivalent.

Data are expressed as mean (SD), percent (n), or median (interquartile range), where appropriate. *P* values were estimated by t-test for continuous variables and chi-square test for categorical variables respectively.

^a^*P* values were estimated using ln-transformed data.

**Supplementary Table S3.** Associations of lower lipid and lipoprotein profiles at baseline with incident EDS when EDS was defined as a CES-D score ≥21 or self-reported use of antidepressant medications

| **Lipid and lipoprotein profile** | **SD** | **HR (95% CI)** | ***P*** |
| --- | --- | --- | --- |
| Conventional lipid measure |  |  |  |
| Total cholesterol, mg d1^-1^ | 34.2 | 1.18 (1.09-1.27) | <0.001 |
| LDL cholesterol, mg d1^-1^ | 30.9 | 1.16 (1.08-1.24) | <0.001 |
| HDL cholesterol, mg d1^-1^ | 14.8 | 0.94 (0.87-1.02) | 0.13 |
| Non-HDL cholesterol, mg d1^-1^ | 34.0 | 1.17 (1.09-1.27) | <0.001 |
| Triglycerides, mg d1^-1^ | 64.6 | 0.97 (0.89-1.03) | 0.25 |
| Total/HDL cholesterol ratio | 1.17 | 1.19 (1.09-1.31) | <0.001 |
| LDL/HDL cholesterol ratio | 0.93 | 1.17 (1.08-1.27) | <0.001 |
|  |  |  |  |
| Lipoprotein particle concentration |  |  |  |
| VLDL-P, nmol l^-1^ |  |  |  |
| Total | 35.2 | 1.04 (0.93-1.15) | 0.51 |
| Large | 5.4 | 1.07 (0.93-1.23) | 0.34 |
| Medium | 21.2 | 1.03 (0.93-1.15) | 0.58 |
| Small | 19.5 | 1.01 (0.94-1.09) | 0.78 |
| IDL-P, nmol l^-1^ | 94.8 | 0.98 (0.90-1.07) | 0.72 |
| LDL-P, nmol l^-1^ |  |  |  |
| Total | 308 | 1.02 (0.92-1.13) | 0.70 |
| Large | 253 | 0.92 (0.83-1.02) | 0.11 |
| Small | 374 | 1.08 (0.97-1.21) | 0.15 |
| HDL-P, µmol l^-1^ |  |  |  |
| Total | 6.6 | 0.92 (0.83-1.03) | 0.15 |
| Large | 3.4 | 0.89 (0.75-1.06) | 0.18 |
| Medium | 6.8 | 1.01 (0.93-1.09) | 0.84 |
| Small | 5.6 | 0.96 (0.89-1.04) | 0.30 |
| Lipoprotein particle size, nm |  |  |  |
| VLDL-P | 7.7 | 1.01 (0.92-1.12) | 0.79 |
| LDL-P | 0.5 | 0.93 (0.84-1.03) | 0.16 |
| HDL-P | 0.5 | 0.94 (0.84-1.04) | 0.21 |

Abbreviations: CES-D, Center for Epidemiological Studies Depression Scale; EDS, elevated depressive symptoms; HDL, high-density lipoprotein; HDL-P, high-density lipoprotein particle; HR, hazards ratio; IDL-P, intermediate-density lipoprotein particle; LDL, low-density lipoprotein; LDL-P, low-density lipoprotein particle; VLDL-P, very-low-density lipoprotein particle.

HR is expressed in terms of per SD decrease in each lipid measure.

Data were adjusted for age, age squared, sex (as both time-independent and dependent variables), race/ethnicity, education, smoking, pack-years of smoking, total gross family income, marital status, any lipid-lowering medication (yes or no), body mass index, heart rate, hypertension, self-reported cancer, interleukin-6, estimated glomerular filtration rate, HDL cholesterol (except for total/HDL cholesterol ratio and LDL/HDL cholesterol ratio), LDL cholesterol (except for total cholesterol, non-HDL cholesterol, total/HDL cholesterol ratio and LDL/HDL cholesterol ratio) and triglycerides, where appropriate.

**Supplementary Table S4.** Associations of lower lipid and lipoprotein profiles at baseline with incident EDS when EDS was defined as a CES-D score ≥16 only

| **Lipid and lipoprotein profile** | **SD** | **HR (95% CI)** | ***P*** |
| --- | --- | --- | --- |
| Conventional lipid measure |  |  |  |
| Total cholesterol, mg d1^-1^ | 34.4 | 1.09 (1.01-1.17) | 0.03 |
| LDL cholesterol, mg d1^-1^ | 31.0 | 1.08 (1.01-1.15) | 0.03 |
| HDL cholesterol, mg d1^-1^ | 14.7 | 1.03 (0.95-1.12) | 0.43 |
| Non-HDL cholesterol, mg d1^-1^ | 34.2 | 1.09 (1.01-1.17) | 0.03 |
| Triglycerides, mg d1^-1^ | 64.8 | 1.03 (0.95-1.11) | 0.51 |
| Total/HDL cholesterol ratio | 1.18 | 1.07 (0.98-1.17) | 0.13 |
| LDL/HDL cholesterol ratio | 0.93 | 1.06 (0.98-1.14) | 0.12 |
|  |  |  |  |
| Lipoprotein particle concentration |  |  |  |
| VLDL-P, nmol l^-1^ |  |  |  |
| Total | 35.3 | 1.01 (0.91-1.12) | 0.86 |
| Large | 5.5 | 0.98 (0.86-1.12) | 0.79 |
| Medium | 21.4 | 1.02 (0.92-1.13) | 0.72 |
| Small | 19.4 | 1.00 (0.93-1.07) | 0.99 |
| IDL-P, nmol l^-1^ | 95.1 | 1.00 (0.92-1.09) | 0.97 |
| LDL-P, nmol l^-1^ |  |  |  |
| Total | 309 | 1.01 (0.92-1.12) | 0.82 |
| Large | 254 | 0.93 (0.85-1.03) | 0.18 |
| Small | 376 | 1.06 (0.96-1.18) | 0.26 |
| HDL-P, µmol l^-1^ |  |  |  |
| Total | 6.6 | 0.99 (0.90-1.11) | 0.92 |
| Large | 3.4 | 0.88 (0.75-1.03) | 0.12 |
| Medium | 6.8 | 0.98 (0.91-1.06) | 0.58 |
| Small | 5.7 | 1.04 (0.96-1.12) | 0.33 |
| Lipoprotein particle size, nm |  |  |  |
| VLDL-P | 7.8 | 1.01 (0.92-1.12) | 0.83 |
| LDL-P | 0.5 | 0.97 (0.88-1.08) | 0.59 |
| HDL-P | 0.5 | 0.93 (0.84-1.03) | 0.17 |

Abbreviations: CES-D, Center for Epidemiological Studies Depression Scale; EDS, elevated depressive symptoms; HDL, high-density lipoprotein; HDL-P, high-density lipoprotein particle; HR, hazards ratio; IDL-P, intermediate-density lipoprotein particle; LDL, low-density lipoprotein; LDL-P, low-density lipoprotein particle; VLDL-P, very-low-density lipoprotein particle.

HR is expressed in terms of per SD decrease in each lipid measure.

Data were adjusted for age, age squared, sex (as both time-independent and dependent variables), race/ethnicity, education, smoking, pack-years of smoking, total gross family income, marital status, any lipid-lowering medication (yes or no), body mass index, heart rate, hypertension, self-reported cancer, interleukin-6, estimated glomerular filtration rate, HDL cholesterol (except for total/HDL cholesterol ratio and LDL/HDL cholesterol ratio), LDL cholesterol (except for total cholesterol, non-HDL cholesterol, total/HDL cholesterol ratio and LDL/HDL cholesterol ratio) and triglycerides, where appropriate.

**Supplementary Table S5.** Subgroup analysis for the associations of lower total, LDL and non-HDL cholesterol concentrations at baseline with incident EDS risk

| **Sub-group** | **n** | **Incident EDS, %** | **Total cholesterol** | | **LDL cholesterol** | | **Non-HDL cholesterol** | |
| --- | --- | --- | --- | --- | --- | --- | --- | --- |
|  |  |  | **HR (95% CI)** | ***P*** | **HR (95% CI)** | ***P*** | **HR (95% CI)** | ***P*** |
| Age, years |  |  |  |  |  |  |  |  |
| 45-54 | 1389 | 27.0 | 1.04 (0.92-1.18) | 0.50 | 1.04 (0.93-1.16) | 0.50 | 1.04 (0.92-1.18) | 0.50 |
| 55-64 | 1410 | 23.8 | 1.12 (0.99-1.27) | 0.08 | 1.11 (0.99-1.24) | 0.08 | 1.12 (0.99-1.27) | 0.08 |
| 65-74 | 1484 | 21.8 | 1.21 (1.07-1.39) | 0.004 | 1.19 (1.06-1.35) | 0.004 | 1.22 (1.07-1.39) | 0.004 |
| 75-84 | 655 | 22.1 | 1.19 (0.97-1.46) | 0.10 | 1.17 (0.97-1.41) | 0.11 | 1.19 (0.97-1.46) | 0.10 |
| *P* for interaction |  |  |  | 0.52 |  | 0.31 |  | 0.33 |
|  |  |  |  |  |  |  |  |  |
| Sex |  |  |  |  |  |  |  |  |
| Men | 2429 | 19.6 | 1.15 (1.04-1.29) | 0.009 | 1.14 (1.03-1.25) | 0.01 | 1.15 (1.04-1.28) | 0.009 |
| Women | 2509 | 28.2 | 1.09 (0.99-1.19) | 0.07 | 1.08 (0.99-1.17) | 0.07 | 1.09 (0.99-1.19) | 0.07 |
| *P* for interaction |  |  |  | 0.12 |  | 0.28 |  | 0.07 |
|  |  |  |  |  |  |  |  |  |
| Race/ethnicity |  |  |  |  |  |  |  |  |
| Caucasian | 1925 | 25.2 | 1.10 (0.98-1.23) | 0.10 | 1.09 (0.98-1.21) | 0.10 | 1.10 (0.98-1.23) | 0.10 |
| African American | 1399 | 28.5 | 1.20 (1.05-1.37) | 0.007 | 1.18 (1.05-1.33) | 0.007 | 1.20 (1.05-1.37) | 0.007 |
| Hispanic American | 976 | 19.4 | 1.08 (0.94-1.24) | 0.26 | 1.07 (0.95-1.22) | 0.26 | 1.08 (0.94-1.24) | 0.26 |
| Chinese American | 638 | 20.8 | 1.13 (0.90-1.42) | 0.29 | 1.11 (0.91-1.37) | 0.31 | 1.13 (0.90-1.42) | 0.29 |
| *P* for interaction |  |  |  | 0.78 |  | 0.75 |  | 0.79 |
|  |  |  |  |  |  |  |  |  |
| CES-D score |  |  |  |  |  |  |  |  |
| Tertile 1 (0-2) | 1564 | 13.9 | 1.24 (1.05-1.47) | 0.01 | 1.21 (1.05-1.41) | 0.01 | 1.24 (1.05-1.46) | 0.01 |
| Tertile 2 (3-6) | 1735 | 20.7 | 1.10 (0.97-1.25) | 0.14 | 1.09 (0.97-1.22) | 0.15 | 1.10 (0.97-1.25) | 0.14 |
| Tertile 3 (7-15) | 1639 | 36.7 | 1.11 (1.01-1.22) | 0.03 | 1.10 (1.01-1.20) | 0.03 | 1.11 (1.01-1.22) | 0.03 |
| *P* for interaction |  |  |  | 0.43 |  | 0.64 |  | 0.82 |
|  |  |  |  |  |  |  |  |  |
| BMI, kg m^-2^ |  |  |  |  |  |  |  |  |
| <25 (< normal) | 1463 | 22.8 | 1.10 (0.96-1.26) | 0.16 | 1.09 (0.97-1.23) | 0.16 | 1.10 (0.96-1.26) | 0.16 |
| 25.0-29.9 (overweight) | 1950 | 23.3 | 1.23 (1.10-1.37) | <0.001 | 1.20 (1.09-1.33) | <0.001 | 1.23 (1.10-1.37) | <0.001 |
| ≥30.0 (obese) | 1525 | 25.5 | 1.05 (0.93-1.18) | 0.42 | 1.05 (0.94-1.16) | 0.41 | 1.05 (0.93-1.18) | 0.42 |
| *P* for interaction |  |  |  | 0.15 |  | 0.23 |  | 0.09 |
|  |  |  |  |  |  |  |  |  |
| Total cholesterol, mg d1^-1^ |  |  |  |  |  |  |  |  |
| <200 (desirable) | 2960 | 24.0 | 1.09 (0.95-1.26) | 0.22 | 1.08 (0.95-1.23) | 0.22 | 1.09 (0.95-1.25) | 0.22 |
| 200-239 (borderline high) | 1532 | 23.5 | 1.53 (1.08-2.17) | 0.02 | 1.47 (1.08-2.02) | 0.02 | 1.53 (1.08-2.16) | 0.02 |
| ≥240 (high) | 446 | 24.0 | 0.92 (0.65-1.30) | 0.64 | 0.93 (0.68-1.27) | 0.65 | 0.92 (0.65-1.30) | 0.64 |
| *P* for interaction |  |  |  | 0.10 |  | 0.33 |  | 0.46 |
|  |  |  |  |  |  |  |  |  |
| LDL cholesterol, mg d1^-1^ |  |  |  |  |  |  |  |  |
| <100 (optimal) | 1404 | 25.4 | 1.07 (0.82-1.39) | 0.64 | 1.06 (0.83-1.34) | 0.65 | 1.07 (0.82-1.39) | 0.47 |
| 100-129 (near optimal/above optimal) | 1938 | 24.3 | 1.37 (0.94-2.01) | 0.10 | 1.33 (0.94-1.88) | 0.10 | 1.37 (0.94-2.00) | 0.10 |
| ≥130 (>high) | 1596 | 22.0 | 1.09 (0.90-1.33) | 0.38 | 1.08 (0.91-1.30) | 0.38 | 1.09 (0.90-1.33) | 0.38 |
| *P* for interaction |  |  |  | 0.27 |  | 0.53 |  | 0.85 |
|  |  |  |  |  |  |  |  |  |
| HDL cholesterol, mg d1^-1^ |  |  |  |  |  |  |  |  |
| <40 (low) | 1142 | 26.4 | 1.09 (0.95-1.25) | 0.20 | 1.09 (0.96-1.23) | 0.18 | 1.10 (0.96-1.26) | 0.19 |
| 40-59 | 2698 | 23.6 | 1.17 (1.06-1.28) | 0.001 | 1.16 (1.06-1.26) | 0.001 | 1.17 (1.07-1.29) | 0.001 |
| ≥60 (high) | 1098 | 21.9 | 1.01 (0.87-1.18) | 0.89 | 1.01 (0.88-1.16) | 0.86 | 1.02 (0.87-1.18) | 0.85 |
| *P* for interaction |  |  |  | 0.57 |  | 0.32 |  | 0.57 |
|  |  |  |  |  |  |  |  |  |
| Non-HDL cholesterol, mg d1^-1^ |  |  |  |  |  |  |  |  |
| <130 (optimal) | 1812 | 25.4 | 1.01 (0.82-1.25) | 0.92 | 1.01 (0.83-1.22) | 0.95 | 1.01 (0.82-1.25) | 0.92 |
| 130-159 (near optimal) | 1721 | 23.6 | 1.25 (0.82-1.91) | 0.29 | 1.23 (0.84-1.80) | 0.28 | 1.25 (0.82-1.91) | 0.29 |
| ≥160 (high) | 1405 | 22.1 | 1.09 (0.90-1.33) | 0.37 | 1.08 (0.91-1.29) | 0.37 | 1.09 (0.90-1.33) | 0.37 |
| *P* for interaction |  |  |  | 0.63 |  | 0.57 |  | 0.60 |
|  |  |  |  |  |  |  |  |  |
| Triglycerides, mg d1^-1^ |  |  |  |  |  |  |  |  |
| <150 (normal) | 3570 | 23.6 | 1.13 (1.04-1.22) | 0.003 | 1.12 (1.04-1.21) | 0.003 | 1.13 (1.04-1.22) | 0.003 |
| 150-199 (borderline high) | 730 | 24.5 | 1.06 (0.89-1.26) | 0.53 | 1.06 (0.90-1.24) | 0.50 | 1.06 (0.89-1.26) | 0.63 |
| ≥200 (>high) | 638 | 24.8 | 1.08 (0.92-1.28) | 0.34 | 1.12 (0.96-1.30) | 0.14 | 1.08 (0.92-1.28) | 0.34 |
| *P* for interaction |  |  |  | 0.57 |  | 0.52 |  | 0.58 |
|  |  |  |  |  |  |  |  |  |
| Total/HDL cholesterol ratio |  |  |  |  |  |  |  |  |
| Tertile 1 (<3.45) | 1645 | 26.7 | 1.09 (0.94-1.28) | 0.26 | 1.08 (0.94-1.25) | 0.27 | 1.09 (0.94-1.28) | 0.26 |
| Tertile 2 (3.45-4.45) | 1646 | 22.7 | 1.31 (1.00-1.73) | 0.05 | 1.28 (1.00-1.65) | 0.05 | 1.31 (1.00-1.73) | 0.05 |
| Tertile 3 (≥4.46) | 1647 | 22.2 | 1.12 (0.96-1.32) | 0.14 | 1.11 (0.96-1.28) | 0.15 | 1.12 (0.96-1.31) | 0.14 |
| *P* for interaction |  |  |  | 0.88 |  | 0.67 |  | 0.69 |
|  |  |  |  |  |  |  |  |  |
| LDL/HDL cholesterol ratio |  |  |  |  |  |  |  |  |
| Tertile 1 (<2.03) | 1655 | 26.6 | 1.07 (0.91-1.27) | 0.40 | 1.06 (0.92-1.24) | 0.41 | 1.07 (0.91-1.26) | 0.40 |
| Tertile 2 (2.03-2.81) | 1632 | 23.0 | 1.32 (0.94-1.85) | 0.11 | 1.28 (0.94-1.75) | 0.11 | 1.31 (0.94-1.85) | 0.11 |
| Tertile 3 (≥2.82) | 1651 | 21.9 | 1.16 (0.97-1.39) | 0.10 | 1.14 (0.97-1.34) | 0.11 | 1.16 (0.97-1.39) | 0.10 |
| *P* for interaction |  |  |  | 0.42 |  | 0.56 |  | 0.33 |

Abbreviations: BMI, body mass index; CES-D, Center for Epidemiological Studies Depression Scale; EDS, elevated depressive symptoms; HDL, high-density lipoprotein; HR, hazards ratio; LDL, low-density lipoprotein.

HR is expressed in terms of per SD decrease in each lipid measure.

HR and *P* values were adjusted for age (except in age-specific analysis), age squared (except in age-specific analysis), sex (as both time-independent and dependent variables, except in sex-specific analysis), race/ethnicity (except in race/ethnicity-specific analysis), education, smoking, pack-years of smoking, total gross family income, marital status, any lipid-lowering medication (yes or no), BMI (except in BMI-specific analysis), heart rate, hypertension, self-reported cancer, interleukin-6, and estimated glomerular filtration rate, HDL cholesterol (except for HDL cholesterol-specific analysis), and triglycerides (except for triglycerides-specific analysis).

**Supplementary Table S6.** Associations of smaller changes in conventional lipid measures with subsequent risk of incident EDS after excluding participants taking statins at visits 1, 2, and/or 3 (n=2824)

| **Conventional lipid measure** | **SD** | **HR (95% CI)** | ***P*** |
| --- | --- | --- | --- |
| Absolute change, mg d1^-1^ |  |  |  |
| Total cholesterol | 23.4 | 0.86 (0.77-0.96) | 0.007 |
| LDL cholesterol | 21.5 | 0.87 (0.78-0.98) | 0.02 |
| HDL cholesterol | 8.3 | 1.02 (0.92-1.14) | 0.68 |
| Non-HDL cholesterol | 22.8 | 0.85 (0.76-0.95) | 0.003 |
| Triglycerides | 50.1 | 0.92 (0.82-1.02) | 0.12 |
|  |  |  |  |
| Relative change, % |  |  |  |
| Total cholesterol | 12.3 | 0.86 (0.77-0.96) | 0.006 |
| LDL cholesterol | 22.0 | 0.94 (0.87-1.01) | 0.10 |
| HDL cholesterol | 15.9 | 1.02 (0.91-1.14) | 0.72 |
| Non-HDL cholesterol | 17.4 | 0.89 (0.81-0.98) | 0.02 |
| Triglycerides | 42.7 | 0.90 (0.81-1.00) | 0.047 |

Abbreviations: BMI, body mass index; EDS, elevated depressive symptoms; HDL, high-density lipoprotein; HR, hazards ratio; LDL, low-density lipoprotein.

HR is expressed in terms of per SD decrease in the change of each lipid measure from baseline visit 1 to visit 3.

Data were adjusted for age, age squared, sex (as both time-independent and dependent variables), race/ethnicity, education, smoking, pack-years of smoking, total gross family income, marital status, body mass index, heart rate, hypertension, self-reported cancer, interleukin-6, estimated glomerular filtration rate, corresponding lipid levels at baseline visit 1, HDL cholesterol, LDL cholesterol (except for total cholesterol and non-HDL cholesterol) and triglycerides at visit 1, history of lipid-lowering medication usage at visits 1 and 3 (“not use at both visits”, “use at visit 1, but not at visit 3”, “not use at visit 1, but use at visit 3” and “use at both visits”), time between visits 1 and 3, and change in weight from visit 1 to visit 3.
